# Short-Term Effects of Healthy Eating Pattern Cycling on Cardiovascular Disease Risk Factors: Pooled Results from Two Randomized Controlled Trials

**DOI:** 10.3390/nu10111725

**Published:** 2018-11-10

**Authors:** Lauren E. O’Connor, Jia Li, R. Drew Sayer, Jane E. Hennessy, Wayne W. Campbell

**Affiliations:** 1 Department of Nutrition Science, Purdue University, West Lafayette, IN 47907, USA; leoconno@purdue.edu (L.E.O.); jiali@uabmc.edu (J.L.); drew.sayer@ucdenver.edu (R.D.S.); janee.hennessy@gmail.com (J.E.H.); 2 Department of Physical Medicine and Rehabilitation, University of Alabama at Birmingham, Birmingham, AL 35233, USA; 3 Anschutz Health and Wellness Center and Division of Endocrinology, Metabolism and Diabetes, University of Colorado Anschutz Medical Campus, University of Colorado, Aurora, CO 80045 USA

**Keywords:** healthy eating pattern, dietary cycling, Mediterranean-style eating pattern, Dietary Approaches to Stop Hypertension eating patter, dietary guidance, overweight and obese adults

## Abstract

Adherence to healthy eating patterns (HEPs) is often short-lived and can lead to repetitive attempts of adopting—but not maintaining—HEPs. We assessed effects of adopting, abandoning, and readopting HEPs (HEP cycling) on cardiovascular disease risk factors (CVD-RF). We hypothesized that HEP cycling would improve, worsen, and again improve CVD-RF. Data were retrospectively pooled for secondary analyses from two randomized, crossover, controlled feeding trials (*n* = 60, 52 ± 2 years, 30.6 ± 0.6 kg/m^2^) which included two 5–6 week HEP interventions (Dietary Approaches to Stop Hypertension-style or Mediterranean-style) separated by a four-week unrestricted eating period. Ambulatory and fasting blood pressures (BP), fasting serum lipids, lipoproteins, glucose, and insulin were measured before and during the last week of HEP interventions. Fasting systolic BP and total cholesterol decreased (−6 ± 1 mm Hg and −19 ± 3 mg/dL, respectively, *p* < 0.05), returned to baseline, then decreased again (−5 ± 1 mm Hg and −13 ± 3 mg/dL, respectively, *p* < 0.05) when adopting, abandoning, and readopting a HEP; magnitude of changes did not differ. Ambulatory and fasting diastolic BP and high-density lipoprotein cholesterol concentrations followed similar patterns; glucose and insulin remained unchanged. Low-density lipoprotein cholesterol concentrations decreased with initial adoption but not readoption (−13 ± 3 and −6 ± 3, respectively, interaction *p* = 0.020). Healthcare professionals should encourage individuals to consistently consume a HEP for cardiovascular health but also encourage them to try again if a first attempt is unsuccessful or short-lived.

## 1. Introduction

Dietary Approaches to Stop Hypertension-style (DASH Pattern) and Mediterranean-style (MED Pattern) eating patterns are considered “healthy eating patterns” (HEPs) among others [1,2,3]. The DASH and MED HEPs are high in fruits, vegetables, whole grains, and lean protein sources and limited in full-fat dairy products, red meats, and refined grains [3,4]. The DASH Pattern is a well-established non-pharmacological approach to reduce blood pressure and also improves total and low-density lipoprotein (LDL) cholesterol concentrations [5]. Adherence to a MED pattern is consistently associated with reduced risk of cardiovascular disease (CVD) and related mortality [6,7] and improves CVD risk factors [8].

Adhering to HEPs promotes cardiovascular health, but long-term HEP adherence is poor, often short-lived, and can lead to repetitive attempts of adopting—but not successfully maintaining—HEPs [9,10,11]. While CVD risk factors are responsive to short-term dietary changes [12,13,14,15,16], it is unknown how repeated cycles of adopting, abandoning, and readopting recommended HEPs (i.e., HEP cycling) influence cardiovascular health. Investigating HEP cycling is warranted, in part, due to possible similarities to weight cycling previously shown to increase CVD risk and related-mortality [17,18,19]. In a novel approach to assess the effects of HEP cycling on CVD risk factors, a secondary analysis was conducted by pooling data from two randomized crossover controlled feeding trials that included a four-week washout period between two HEP interventions: the DASH Study [20] and the MED Study [21]. We hypothesized that (1) adopting HEPs would improve CVD risk factors, (2) abandoning HEPs would reverse improvements in CVD risk factors from initial HEP adoption, and (3) the magnitude of changes in CVD risk factors would not differ between initial and subsequent HEP adoption.

## 2. Materials and Methods

### 2.1. Experimental Design

**Figure 1:**
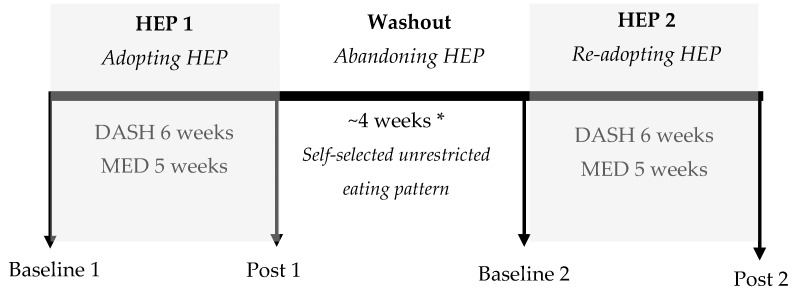
Study schematic of assessing effects of healthy eating pattern cycling on cardiovascular disease risk factors. Cardiovascular disease risk factors were measured at baseline 1, post 1, baseline 2, and post 2. Participants consumed self-selected foods during baseline 1, throughout the washout period, and during baseline 2. Participants consumed prescribed healthy eating patterns during post 1 and post 2. HEP, healthy eating pattern; DASH, Dietary Approaches to Stop Hypertension-style eating pattern; MED, Mediterranean-style eating pattern; * median = 28 days.

A priori, the DASH Study [20] and MED Study [21] assessed effects of consuming HEPs containing varying amounts of lean minimally processed red meat on CVD risk factors. The crossover experimental design allows for novel investigation of chronological effects of HEP cycling, rather than randomized treatment effects of red meat consumption, on CVD disease risk factors. Therefore, the amounts of lean unprocessed red meat consumed were randomly distributed between HEP intervention 1 and HEP intervention 2. In the DASH Study [20], 19 participants were randomly assigned to consume a DASH Pattern with 55% of total protein as pork tenderloin or lean chicken/fish for six weeks. Participants then consumed their unrestricted self-selected eating pattern during a four-week washout period and consumed the alternative DASH Pattern for another six weeks. In the MED Study [21], 41 participants were randomly assigned to consume a MED Pattern with either 200 g or 500 g of beef/pork tenderloin for five weeks, consumed their unrestricted self-selected eating pattern for four weeks, and then consumed the alternative MED Pattern for another five weeks. Cardiovascular disease risk factors were measured at baseline 1 and 2 (while participants were consuming their unrestricted self-selected eating pattern) and during the last week of each HEP intervention (post 1 and 2); see Figure 1.

### 2.2. Ethics

The Purdue University Biomedical Institutional Review Board approved all study procedures and documents (DASH Study: 1112011665 conducted May 2012–December 2013, and MED Study: 1501015662 conducted July 2015–December 2016). Participants provided written informed consent and received a monetary stipend for participation. Studies were registered at clinicaltrials.gov (DASH Study: NCT01696097 and MED Study: NCT02573129).

### 2.3. Participant Inclusion Criteria

Non-smoking and non-diabetic individuals with normal liver and kidney functions were recruited for both studies from the Greater Lafayette, IN area. Individuals with systolic blood pressure ≥120 mm Hg or diastolic blood pressure ≥80 mm Hg, aged 21–75 years were recruited for the DASH Study. Individuals who were overweight or obese (25–37 kg/m^2^), aged 30–69 years who had total cholesterol <260 mg/dL, LDL cholesterol <190 mg/dL, triglycerides <400 mg/dL, fasting glucose <110 mg/dL, systolic blood pressure <160 mm Hg, and diastolic blood pressure <100 mm Hg were recruited for the MED Study. A physician reviewed all participants’ screening measurements to ensure that they met the inclusion criteria for each study and approved them for participation. Participants in both studies self-reported weight stability (± 4.5 kg) and consistent physical activity levels for at least 3 months prior to starting each study. Participant medication usage was stable for 6 months prior to and throughout each study.

### 2.4. Decsription of Prescribed HEPs

The DASH [20] and MED Patterns [21] consumed by participants were described in detail previously. In brief, both eating patterns were high in fruits, vegetables, whole grains, and lean protein sources (see Table 1). The DASH and MED Patterns contained 17/57/27% and 19/42/40% of total energy from protein/carbohydrate/fat, respectively, and both eating patterns contained <8% of total energy from saturated fat (calculated three-day average during the last week of each intervention). Both HEPs contained ~2700, ~5000, and ~500 mg/day of sodium, potassium, and magnesium, respectively.

About 30% and 100% of the foods prescribed in the DASH Study and MED Study, respectively, were prepared for and provided to participants. For the DASH Study, participants were counseled to purchase, prepare, and consume portioned quantities of non-provided foods. All meats, as well as some snacks and condiments, were provided. Menus were developed (Pronutra, Viocare, Inc. Princeton, NJ, USA) to maintain participant’s baseline 1 body mass, with daily energy intake estimated using sex-specific equations [22]. Participants tracked compliance and reported menu deviations using daily menu check-off lists. Participants met with study staff weekly to pick up food, drop-off menu checkoff lists, and measure body mass. Compliance to the prescribed HEPs was measured from three days of menu check-off lists during the last week of each intervention.

### 2.5. Assessment of Baseline and Prescribed Eating Patterns

In both studies, participants recorded their self-selected unrestricted eating patterns at both baselines using 3-day food records. Researchers retrospectively scored 3-day food records from the DASH Study using the Dixon DASH index [23]. In the MED Study, participants self-completed the Mediterranean Diet Assessment Tool (MEDAS) [24] before being randomized into the study. The prescribed HEP menu representing the median energy content for each study was scored using the appropriate dietary index and are described in Table 1.

The Dixon DASH index [23] is a sex-specific scoring system, based on the 2005 Dietary Guidelines for Americans’ DASH Eating Plan [25], that allocates one point for meeting the following nine requirements: (1) fruit including fruit juice; ≥4 servings/day for men and women, (2) vegetables, including potatoes; ≥4 servings/day for men and ≥3 servings/day for women, (3) whole grains defined as ≥67% whole grain; ≥4.7 servings/day for men and ≥4 servings/day for women, (4) dairy; ≥2 servings/day for men and women, (5) nuts, seeds, and legumes; ≥4 servings/week for men and ≥3 servings/week for women, (6) meat or meat equivalents; <6 oz (170 g)/day for men and women, (7) added sugar intake ≤3% of total energy intake, (8) saturated fat intake ≤5% of total energy intake, and (9) alcoholic drinks; ≤2 drinks/day for men or ≤1 drink/day for women.

The MEDAS [24] is a scoring system that allocates one point for meeting the following 14 requirements: (1) olive oil as main source of fat, (2) olive oil; ≥¼ cup/day, (3) vegetables; ≥3 cups/day, (4) fruit; ≥3 servings/day, (5) red meat or meat products; <2 servings/day, (6) butter; <1 tbsp/day, (7) sweet or carbonated beverages; <1/day, (8) wine; ≥7 glasses/week, (9) cooked legumes; ≥3 cups/week, (10) fish or shellfish; ≥3 servings/week, (11) commercial sweets/pastries; <3 servings/week, (12) nuts; ≥3/4 cup/week, (13) preferential consumption of white meat instead of red meat, and (14) sofrito (tomato, onion, leak, or garlic simmered in olive oil); ≥2 dishes/week.

### 2.6. Outcomes Measured

Cardiovascular disease risk factors were measured chronologically at baseline 1, post 1, baseline 2, and post 2 (Figure 1). Outcomes included: (1) fasting body mass and composition estimated using the BOD POD Gold Standard Body Composition Tracking System (COSMED USA, Inc., Concord, CA, USA); (2) fasting clinical blood pressures (HEM-780, Omron Healthcare, Inc.) measured in a seated position after 15 min of rest; (3) 24-h ambulatory blood pressures (Oscar2, Suntech Medical, Inc., Morrisville, NC, USA) analyzed as total 24-h, waking (0800-2100), and sleeping (2230-0730) periods; (4) fasting serum total, LDL, and high-density lipoprotein (HDL) cholesterol, triglycerides, glucose, and insulin concentrations; and (5) homeostatic model assessment for insulin resistance [(HOMA-IR) = (fasting glucose mg/dL × fasting insulin µIU/mL)/405]. Descriptions of the analytical methods employed were described in more detail previously [20,21].

### 2.7. Statistics

Participants’ data were independently double entered and coded to maintain confidentiality. Participants who completed both HEP interventions were included in analysis. A doubly repeated measures (order of treatment, pre and post intervention) mixed effects ANCOVA was performed using a generalized linear mixed model in SAS (SAS 9.4, SAS Institute Inc., Cary, NC, USA). The model included age, sex, body mass at each time point, study (DASH or MED), randomized treatment (lower vs higher red meat consumption), order of treatment, pre and post intervention time point, and appropriate interaction terms, with a random participant effect. LSMESTIMATE statements were invoked to (1) estimate the main effects of time (post vs. baseline among all participants), (2) compare baseline 1 with baseline 2 to assess the effectiveness of the washout (abandonment) period, and (3) compare the magnitudes of change from baseline to post during HEP 1 vs. HEP 2. Results are presented as least squares (LS) mean ± SE of LS mean unless otherwise stated. Two-tailed significance was set at *p* < 0.05 with Bonferroni adjustment within each outcome variable. The DASH Study and MED Study were previously powered to detect pre to post differences in systolic blood pressure (DASH and MED) and total cholesterol (MED) [20,21]. In this secondary analysis, 60 participants provided >80% and >95% power to detect an effect size of 0.4 and 0.5 (Cohen’s d), respectively, between HEP 1 and HEP 2 values if a difference was present [26].

## 3. Results

### 3.1. Participants

Sixty participants (41 female, 19 male, predominantly Caucasian) were included in this study (see Figure S1 for CONSORT diagram). Participant baseline 1 characteristics are shown in Table 2.

### 3.2. Dietary Intakes

At baseline 1, participants in the DASH Study were not consuming a DASH Pattern (Dixon Index mean score 2 ± 1 out of 9) and participants in the MED Study were not consuming a MED Pattern (MEDAS mean score 4 ± 0 out of 14). Self-reported compliance to the prescribed HEPs was ≥95% in both studies. Dixon Index and MEDAS scores increased by 250% (Dixon Index from 2 to 7 points) for the DASH Study and 225% (MEDAS from 4 to 13 points) for the MED Study during the HEP interventions compared to baseline. For both studies, composition of participants’ unrestricted eating patterns did not differ between baseline 1 and baseline 2, supporting that they resumed their usual self-selected eating patterns during the washout period.

### 3.3. Cardiovascular Disease Risk Factors

#### 3.3.1. Overall HEP Adoption

Adopting HEPs for 5–6 weeks decreased ambulatory (total 24-h, waking, and sleeping) and fasting blood pressures; total, LDL, and HDL cholesterols; insulin and HOMA-IR; and total body mass and percent body fat. Triglycerides, total cholesterol: HDL, and glucose were unchanged (Table S1).

#### 3.3.2. Initial HEP Adoption (HEP 1)

Initial HEP adoption decreased 24-h and waking ambulatory as well as fasting blood pressures; total, LDL, and HDL cholesterols; and total body mass and body fat percentage. Sleeping ambulatory blood pressures, triglycerides, total cholesterol: HDL, glucose, insulin, and HOMA-IR were unchanged (Table S1).

#### 3.3.3. HEP Abandonment (Washout)

After abandoning HEPs for four weeks, 24-h and waking ambulatory and fasting blood pressures; total, LDL, and HDL cholesterols; and body fat percentage returned to baseline 1 values (i.e., baseline 1 and baseline 2 values did not differ; Table 2). No other CVD risk factor values differed between baseline 1 and baseline 2 except total body mass was 1.3 ± 1.9 kg higher at baseline 1 than baseline 2.

#### 3.3.4. HEP Readoption (HEP 2)

Readopting HEPs decreased all ambulatory and fasting blood pressures and total and HDL cholesterols. Decreases in these outcomes during initial adoption and readoption phases did not differ in magnitude. Readopting HEPs did not change LDL, triglycerides, total cholesterol: HDL, glucose, insulin, and HOMA-IR. Total body mass decreases were blunted during HEP 2 compared to HEP 1 (−2.2 ± 0.2 during HEP 1, *p* < 0.001 and −1.4 ± 0.2 during HEP 2, *p* < 0.001) but body fat percentage decreased non-differentially (Table S1).

#### 3.3.5. HEP Cycling

**Figure 2:**
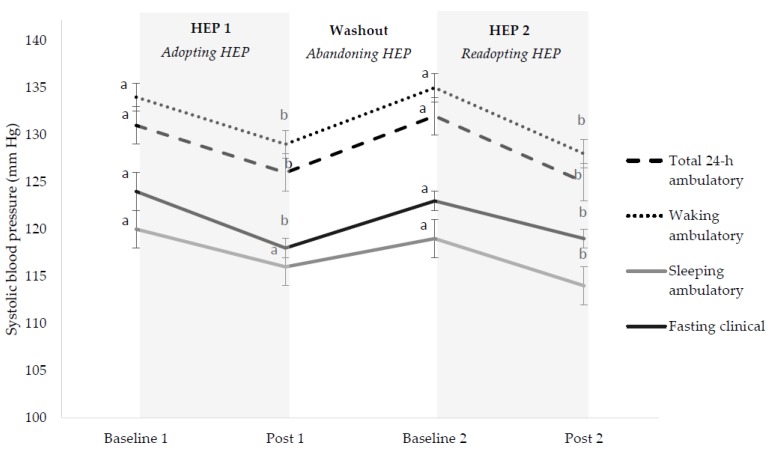
Systolic blood pressure changes during healthy eating pattern cycling. Results are presented as LS mean ± SEM of LS mean, *n* = 60. HEP; healthy eating pattern. Subjects consumed self-selected foods during baseline 1, throughout the washout (abandoning) period, and during baseline 2. Subjects consumed prescribed healthy eating patterns during post 1 and post 2. Different letter superscripts represent different values (*p* < 0.05). Results for diastolic blood pressures followed a similar pattern; see Table S1.

**Figure 3:**
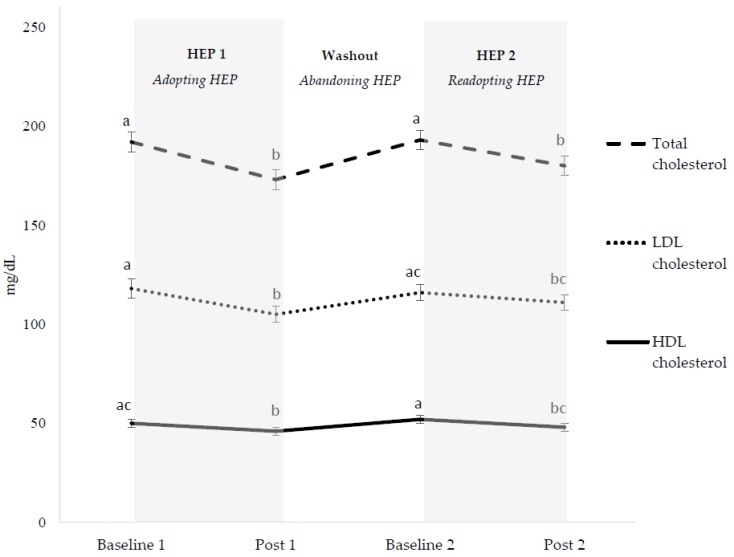
Blood lipid and lipoprotein changes during healthy eating pattern cycling. Results are presented as LS mean ± SEM of LS mean, *n* = 60. HEP; healthy eating pattern. Subjects consumed self-selected foods during baseline 1, throughout the washout (abandoning) period, and during baseline 2. Subjects consumed healthy eating patterns during post 1 and post 2. Different letter superscripts represent different values (*p* < 0.05). Conversion to mmol/L for total, LDL, and HDL cholesterol: multiply mg/dL by 0.0259.

Total 24-h, waking, and fasting blood pressures (Figure 2) and total and HDL cholesterols (Figure 3) decreased, returned to baseline, and decreased again when adopting, abandoning, and readopting HEPs, respectively. Decreases in these outcomes during initial adoption and readoption phases did not differ in magnitude. LDL cholesterol decreased, returned to baseline, and then did not change when adopting, abandoning, and readopting HEPs, respectively (Figure 3). Body mass decreased, increased (but did not return to baseline), and then decreased to a lesser magnitude when adopting, abandoning, and readopting HEPs (Table S1).

## 4. Discussion

These results emphasize that CVD risk factors are sensitive to short-term dietary changes but are the first, to the best of our knowledge, to show that HEP cycling is not detrimental to subsequent improvements in most CVD risk factors. Consuming HEPs, such as a DASH or MED Pattern, improves CVD risk factors within 5–6 weeks, but these improvements are lost within four weeks of abandonment. Individuals can improve most CVD risk factors to a comparable extent by readopting HEPs. Our research emphasizes the importance of consistently consuming HEPs for cardiovascular health.

Our results support previous research showing that consuming a DASH Pattern or MED Pattern improves CVD risk factor profiles. Blood pressure improvements during the DASH and MED HEP interventions (up to −6 and −4 mm Hg for systolic and diastolic blood pressure, respectively) were comparable to the original randomized 8-week DASH [12] and 3-month MED [27] Pattern controlled trials. Reductions in total, LDL, and HDL in our 5–6 week study (−19, −13, and −4 mg/dL, respectively) were comparable to the original 8-week DASH trial [28] (−14, −11, and −4 mg/dL, respectively) and twice the magnitude which occurred in the original three-month MED trial [27] (−5, −6, and −2 mg/dL, respectively). Therefore, HEPs are effective short- and longer-term non-pharmacologic approaches to decrease indicators of CVD risk [3], but additional lifestyle modifications, such as weight loss or exercise may be needed to improve glycemic control [29,30].

Participants’ CVD risk factors returned to baseline values from abandoning HEPs for four weeks, highlighting the importance of sustained HEP consumption. Previous research shows that self-reported adherence to prescribed eating patterns drops ~10% each month with participants being <40% adherent after 6 months of a dietary intervention [31]. Eating behavior is affected by a complex set of behavioral, biological, environmental, and psychosocial factors that makes long-term HEP adherence difficult for most people [32]. Health-related behaviors are also driven largely by routines and habits, and altering those behaviors relies extensively on executive brain function. However, executive functioning is effortful and prone to error, especially in difficult environments and/or circumstances [33] such as ready access to inexpensive, energy dense, and palatable foods [34]. Changes in current food environments are needed to ease consistent consumption of HEPs to avoid fluctuations in cardiovascular health.

We observed comparable improvements in CVD risk factors when participants readopted HEPs after a period of self-selected eating, except LDL concentrations. LDL concentrations did not differ between baseline 1 and baseline 2, but decreases were blunted during HEP 2 compared to HEP 1. Similar results occurred in a study designed to assess the effects of weight cycling on CVD risk factors in non-obese young women. During the first and second 4-week energy-restricted periods, LDL concentrations decreased by −18.8 ± 7.7 and −9.2 ± 1.1 mg/dL, respectively, compared to initial baseline values [35]. These results combined with ours indicate that improvements in LDL concentrations were blunted by ~100% during a second attempt to either lose weight or consume a HEP. Interventional studies are needed to assess whether 3+ repeated cycles of HEP cycling further inhibit improvements in LDL concentrations as well as long-term consequences of repeated HEP cycles on CVD onset and related events.

Weight cycling commonly causes individuals to regain more body mass than initially lost, causing an upward “drift” in baseline body mass [18,36]. The current results show a downward baseline body mass drift during one HEP cycle with a prescribed four-week period of HEP abandonment. During the washout period, participants regained about half of the body mass initially lost, resulting in a lower value at baseline 2 than baseline 1. This downward “drift” in body mass likely explains the blunted body mass decrease observed when participants readopted HEPs compared to initial adoption. It is unclear if blunted decreases in body mass were related to blunted decreases in LDL during HEP 2. Exploratory post hoc assessments revealed that changes in body mass were correlated with changes in LDL (*r*^2^ = 0.10, *p* = 0.0036) and total cholesterol (*r*^2^ = 0.10, *p* = 0.0010) but not correlated with other CVD risk factors. There was no difference in the magnitude of body mass loss between the two energy restricted periods in the study mentioned previously that showed similar patterns in LDL changes [35]. Therefore, caution is warranted with regard to whether the blunted LDL response during HEP 2 may be explained by changes in body mass.

Data for this secondary analysis were pooled from two randomized crossover controlled feeding trials [20,21] in which each participant consumed prescribed HEPs during two separate intervention periods. The dietary control achieved may be considered a strength and a limitation. Tightly controlled short-term feeding trials in which some or all of the foods are provided to participants allow researchers to assess the efficacy of eating patterns on cardiovascular health. However, adherence to eating patterns is lower when participants are counseled on what foods to consume compared to when food is provided [37]. Therefore, changes in CVD risk factors may not be as apparent if participants were not prescribed menus and provided foods. The present study was limited by measuring CVD risk factors at only four time points. In the original DASH trial, blood pressures dropped after two weeks of participants consuming a DASH Pattern and then stabilized for the remaining eight-week intervention [12]. Future studies should measure CVD risk factors more frequently to assess when changes occur during HEP cycling. Future research should also consider timing and frequency of HEP consumption, as higher meal/snack frequency is associated with better cardiovascular health [38].

## 5. Conclusions

Our study demonstrates the importance of consistently consuming HEPs, as CVD risk factors are sensitive to short-term dietary changes. Readopting HEPs after a period of unhealthy eating apparently does not hinder the responsiveness of CVD risk factors, except possibly LDL, to improve. Future research should address the potential impact of repeated HEP cycling on metabolic responsiveness, including changes in lipids and lipoproteins, and progression toward CVD development. Health practitioners and future public policies should focus on strategies to help individuals sustain HEPs as a lifestyle modification to avoid fluctuations in cardiovascular health.

## Figures and Tables

**Table 1:** Prescribed daily and weekly food group servings for the median energy intake level ^a^.

	DASH Study	MED Study
Fruits (servings/day ^b^)	7	4
Vegetables (servings/day ^c^)	8	6
Dark green vegetables	1	1
Red and orange vegetables	2	1
Legumes	1	1
Starchy vegetables	2	1
Other vegetables	2	3
Whole grains (servings/day ^d^)	5	4
Protein-rich foods (g/week ^e^)		
Red meat	422	336
Poultry	299	266
Seafood	185	336
Whole eggs	0	3
Nuts, seeds, soy ^f^	80	588
Dairy (servings/day ^g^)	4	3
Olive oil (tsp/week ^h^)	n/a	55
Corresponding diet index score	7 out of 9 total points ^i^	13 out of 14 total points ^j^

^a^ food group servings averaged across a 7 day menu cycle for median 2400 kcal menu, ^b^ ½ cup or 1 medium fresh fruit, ^c^ ½ cup fresh or 1 cup leafy, ^d^ 28 g= ½ cup or 1 oz, ^e^ 28 g= 1 oz; cooked weights, ^f^ 28 g = 1 tbsp. nut butter or 1/2 oz nuts/seeds or ~1 oz-equivalent, ^g^ 1 cup milk or yogurt, ^h^ 4.5g= 1 tsp., ^i^ Dixon DASH Index(19), ^j^ Mediterranean Diet Assessment Tool(20).

**Table 2:** Subject baseline 1 fasting cardiovascular health profiles.

Outcome	DASH(*n* = 19)	MED(*n* = 41)	All(*n* = 60)
Age (years)	61 ± 2	46 ± 2	52 ± 2
BMI (kg/m^2^)	31.0 ± 1.4	30.5 ± 0.6	30.6 ± 0.6
Fasting systolic BP (mm Hg)	130 ± 2	118 ± 2	121 ± 2
Fasting diastolic BP (mm Hg)	85 ± 2	80 ± 1	82 ± 1
Total cholesterol (mg/dL)	199 ± 9	192 ± 5	195 ± 4
Low-density lipoprotein (LDL) cholesterol (mg/dL)	120 ± 8	119 ± 4	120 ± 4
High-density lipoprotein (HDL) cholesterol (mg/dL)	54 ± 3	49 ± 2	51 ± 2
Total cholesterol:HDL cholesterol	3.78 ± 0.20	4.18 ± 0.19	4.05 ± 0.15
Triglycerides (mg/dL)	121 ± 12	118 ± 10	119 ± 7
Glucose (mg/dL)	93 ± 2	99 ± 1	97 ± 1
Insulin (µIU/mL)	12.6 ± 2.1	12.4 ± 1.2	12.5 ± 1.1
HOMA-IR	3.005 ± 0.573	3.076 ± 0.324	3.053 ± 0.281
Body mass (kg)	86.4 ± 3.7	87.5 ± 2.6	87.2 ± 2.1
Body fat (%)	41.7 ± 2.1	38.9 ± 1.5	39.8 ± 1.2

Results are presented as mean ± SEM. BP, blood pressure. Conversion factor to SI units are as follows: total, LDL, and HDL cholesterol mmol/L = mg/dL × 0.0259, triglycerides mmol/L= mg/dL × 0.0113, glucose mmol/L= mg/dL × 0.0555, and insulin pmol/L= µIU/mL × 6.945.

## Supplementary Materials

The following are available online at http://www.mdpi.com/2072-6643/10/11/1725/s1, Figure S1: Participant recruitment diagram, Table S1: Changes in cardiovascular disease risk factors during repeated periods of consuming a prescribed healthy eating pattern (HEP).
