# Supplementary material for: Short-Term Effects of Healthy Eating Pattern Cycling on Cardiovascular Disease Risk Factors: Pooled Results from Two Randomized Controlled Trials
Source: Nutrients. 2018 Nov 10;10(11):1725. doi: 10.3390/nu10111725 (PMC6266045; doi:10.3390/nu10111725)
Supplement: Supplementary file 1 [file nutrients-10-01725-s001.pdf]

Figure S1: Participant recruitment diagram

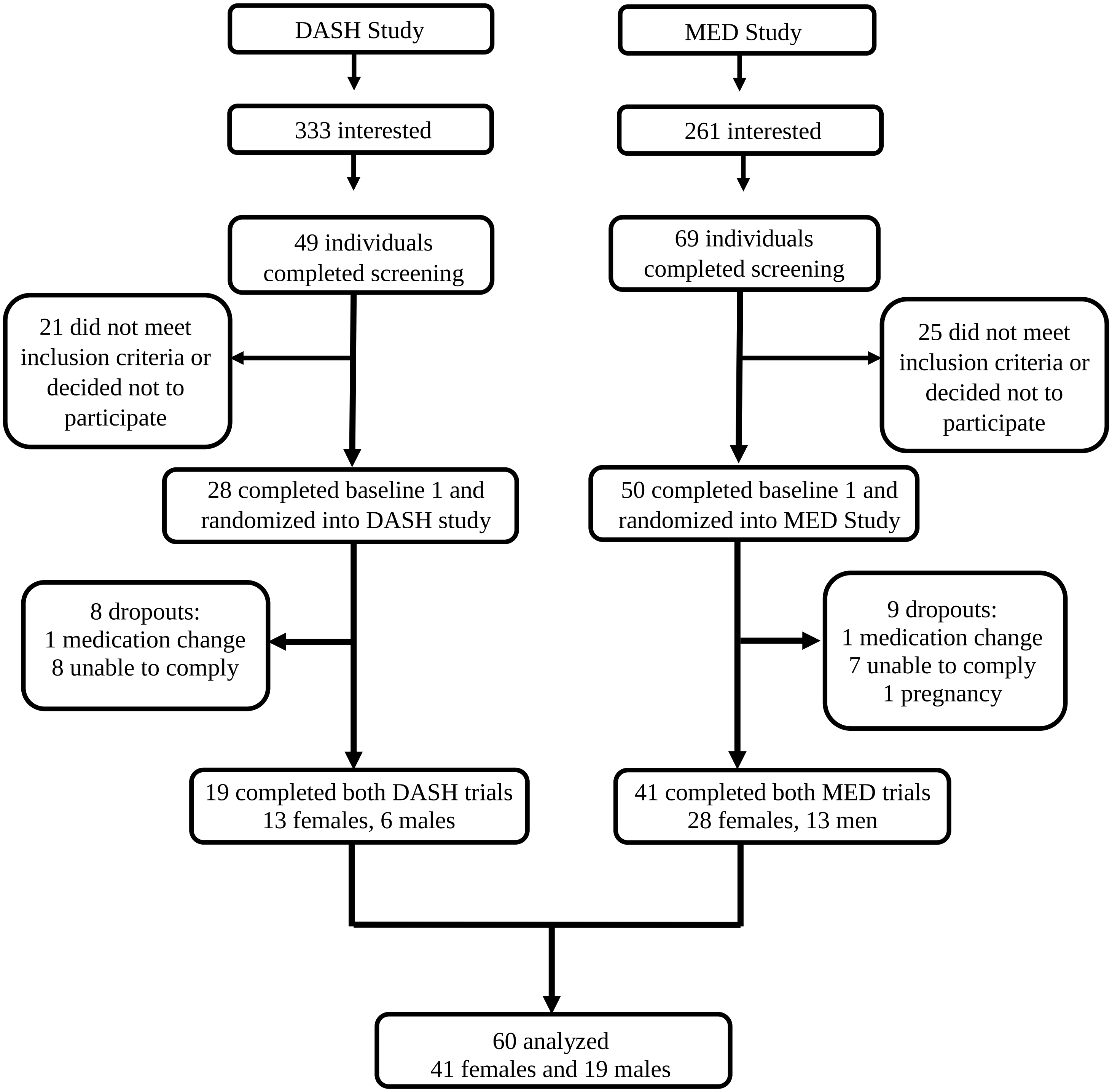

Table S1. Changes in cardiovascular disease risk factors during repeated periods of consuming a prescribed healthy eating pattern (HEP).

	HEP 1 *Adopting HEP*	HEP 2 *Readopting HEP*	HEP adoption p value [1]	HEP order p value [2]
24-hr SBP (mm Hg)	-5 ± 1*	-7 ± 1 *	<0.0001	0.4354
24-hr DBP (mm Hg)	-3 ± 1*	-3 ± 1 *	<0.0001	0.9366
Waking SBP (mm Hg)	-5 ± 1*	-7 ± 2 *	<0.0001	0.7556
Waking DBP (mm Hg)	-4 ± 1*	-4 ± 1 *	<0.0001	0.9903
Sleeping SBP (mm Hg)	-4 ± 2	-5 ± 2 *	0.0005	0.5294
Sleeping DBP (mm Hg)	-2 ± 1	-2 ± 1 *	0.0018	0.3963
Fasting SBP (mm Hg)	-6 ± 1*	-5 ± 1 *	<0.0001	0.9023
Fasting DBP (mm Hg)	-4 ± 1*	-4 ± 1 *	<0.0001	0.6146
Total cholesterol (mg/dL)	-19 ± 3*	-13 ± 3 *	<0.0001	0.0906
LDL cholesterol (mg/dL)	-13 ± 3*[a]	-6 ± 3 [a¥]	<0.0001	0.0202
HDL cholesterol (mg/dL)	-4 ± 1*	-5 ± 1 *	<0.0001	0.6306
Triglycerides (mg/dL)	-7 ± 6	-10 ± 6	0.0505	0.7528
TC:HDL	-0.08 ± 0.08	0.06 ± 0.07	0.8798	0.1398
Glucose (mg/dL)	-1 ± 1	1 ± 1	0.9103	0.3266
Insulin (µIU/mL)	-1.4 ± 1	-2.8 ± 1	0.0037	0.3057
HOMA-IR	-0.388 ± 0.224	-0.621 ± 0.224	0.0024	0.4611
Body mass (kg)	-2.2 ± 0.2*[a]	-1.4 ± 0.2*[b]	<0.0001	0.0013
Body fat (%)	-1.1 ± 0.3*	-1.1 ± 0.3*	<0.0001	0.9432

Results are presented as LS means ± SEM of LS means adjusted for age, sex, body mass at each time point, study, and designated treatment within each study with a random participant effect, *n* = 60. [1] overall time effect of adopting HEP (all data combined during HEP 1 and HEP2) [2] changes from baseline in HEP 1 vs changes from baseline in HEP 2 (time × order interaction), * indicates a HEP-specific change over time assessed when time or time × order *p* value <0.05, different letters indicate different magnitude in changes over time, ¥ indicates trend *p* = 0.061 for difference in magnitude of HEP 1 and HEP 2, Conversion factor to SI units as follows: total, LDL, and HDL cholesterol mmol/L = mg/dL × 0.0259, triglycerides mmol/L= mg/dL × 0.0113, glucose mmol/L = mg/dL × 0.0555, and insulin pmol/L = µIU/mL × 6.945.
